# The anatomy of the inferior mesenteric artery: a systematic review with meta-analysis

**DOI:** 10.1007/s00276-025-03657-1

**Published:** 2025-05-24

**Authors:** George Triantafyllou, Nektarios Belimezakis, Orestis Lyros, Nikolaos Arkadopoulos, Fotis Demetriou, George Tsakotos, Maria Piagkou

**Affiliations:** 1 Department of Anatomy, School of Medicine, Faculty of Health Sciences, National and Kapodistrian University of Athens, 75 Mikras Asias str, Goudi, Athens, 11527 Greece; 2 Fourth Department of Surgery, Attikon University Hospital, National and Kapodistrian University of Athens, Haidari, Greece

**Keywords:** Inferior mesenteric artery, Variation, Meta-analysis, Evidence-based anatomy, Surgical anatomy

## Abstract

**Purpose:**

This evidence-based systematic review with meta-analysis aims to synthesize the inferior mesenteric artery (IMA) variants including their origin level, branching patterns, and morphometric characteristics.

**Materials and methods:**

The study adhered to the latest guidelines. Four online databases were used for the systematic review, and studies that reported the prevalence of IMA variants were considered eligible for inclusion. A statistical meta-analysis was conducted using R programming software with a random-effects model to calculate the pooled prevalence of the variants.

**Results:**

Twenty-three (23) studies were included in this analysis. The IMA typically originated at the 3rd lumbar vertebra (L3) level, occurring in 70.16% of cases. The most common branching pattern of the IMA was bifurcated, indicating a pooled prevalence of 63.89%. This predominant pattern involved the IMA bifurcating into the left colic artery (LCA) and a common trunk for both the superior rectal artery (SRA) and the sigmoid artery (SA), with a pooled prevalence of 46.09%. The IMA trifurcation and tetrafurcation had pooled prevalences of 27.35% and 11.62%, respectively. The diameter of the IMA had a pooled mean of 41.41 mm, and the distance from the IMA’s origin to the LCA had a pooled mean of 40.67 mm.

**Conclusions:**

This review of existing literature delineates the IMA levels of origin, branching patterns, and morphometric characteristics. A comprehensive understanding of the surgical anatomy of this vessel is imperative during colorectal cancer procedures. Consequently, surgeons operating in this anatomical region must possess an in-depth knowledge of typical and variant anatomical structures.

**Supplementary Information:**

The online version contains supplementary material available at 10.1007/s00276-025-03657-1.

## Introduction

Cadaveric, imaging, and surgical studies have continuously investigated the surgical anatomy of abdominal vessels. Evidence-based anatomical meta-analyses offer a thorough summary of significant variants in these vessels that may greatly impact surgical procedures [3–7, 35, 39, 40].

The abdominal aorta (AA) major branches provide an arterial supply to the abdominal organs. These vessels are the coeliac trunk (CeT) and the superior and inferior mesenteric arteries (SMA and IMA). The IMA originates from the AA’s anterior aspect at the third lumbar vertebra level (L3). It is located posterior to the inferior border of the horizontal part of the duodenum [34]. The IMA typically gives off the left colic and sigmoid arteries (LCA and SA). It crosses the origin of the left common iliac artery with the inferior mesenteric vein (IMV) located between the two arteries. Then, it continues as the superior rectal artery (SRA) in the sigmoid mesocolon’s root [34]. The IMA supplies a major portion of the gastrointestinal tract, the so-called hindgut (distal 1/3 of the transverse colon, splenic flexure, descending colon, sigmoid colon, and rectum) [34]. *Bergman’s Comprehensive Encyclopedia of Human Anatomic Variation* describes the IMA variants as relatively rare; however, the typical branching pattern and origin levels are frequently studied [36]. The surgical significance of the IMA anatomy is attributed to its topographical characteristics and branching pattern, as its branches are frequently ligated during colorectal cancer surgery [36].

Two comprehensive reviews of the current literature concerning the IMA variants exist [24, 43]; however, both lack a meta-analytic approach. The evidence-based systematic review, accompanied by a meta-analysis, seeks to elucidate the IMA’s morphology and morphometry. Additionally, the significance of the surgical procedure will be discussed in detail.

## Materials and methods

### Search strategy

The systematic review with meta-analysis was performed according to the Evidence-based Anatomy Workgroup guidelines for anatomical meta-analysis [15] and the PRISMA 2020 guidelines for systematic reviews [30].

A literature search was done on PubMed, Google Scholar, Scopus, and Web of Science online databases. The following terms were used in several combinations: “inferior mesenteric artery,” “variation,” “cadaveric study,” “imaging study,” and “surgical study.” The references of all included articles were evaluated, the grey literature was investigated, and a hands-on search of the significant anatomical journals (*Annals of Anatomy*,* Clinical Anatomy*,* Journal of Anatomy*,* Anatomical Record*,* Surgical and Radiological Anatomy*,* Folia Morphologica*,* European Journal of Anatomy*,* Anatomical Science International*,* Anatomy and Cell Biology*,* Morphologie*) was performed. Inclusion criteria were studies reporting the prevalence of IMA variants. Case reports, conferences’ abstracts, animal studies, and studies that reported irrelevant or insufficient data were excluded.

Two independent reviewers (GTr and NB) performed the literature search and extracted the data into Microsoft Excel sheets. The results were compared, and the other authors resolved potential differences. The Anatomical Quality Assurance (AQUA) tool, created by the Evidence-based Anatomy Workgroup for anatomical systematic reviews [16], was used to evaluate the risk of bias.

### Statistical analysis

A statistical meta-analysis was conducted using the open-source R programming language and RStudio software (version 4.3.2), using the “meta” and “metafor” packages by a single researcher (GTr). The pooled prevalence was calculated using the inverse variance and random effects models. The proportions (prevalence) meta-analysis was conducted using the Freeman-Tukey double arcsine transformation, the DerSimonian-Laird estimator for the between-study variance tau^2^, and the Jackson method for confidence interval of tau^2^ and tau. Moreover, several subgroup analyses were performed to detect variables (geographic distribution or type of study) affecting the estimated pooled prevalence. The means (mean diameter) meta-analysis was conducted using the untransformed (raw) means, the restricted maximum-likelihood estimator for tau^2^, and the Q-Profile method for confidence interval of tau^2^ and tau. A p-value of less than 0.05 was considered statistically significant. Cochran’s Q statistic was used to evaluate the presence of heterogeneity across studies, and the Higgins I^2^ statistic was used to quantify heterogeneity. Cochran’s Q p-value < 0.10 was considered significant. Higgins I^2^ values between 0 and 40% were regarded as not significant, 30–60% as moderate heterogeneity, 50–90% characterized as substantial heterogeneity, and 75–100% may represent considerable heterogeneity [15]. To evaluate the presence of a small-study effect (the phenomenon that smaller studies may show different effects than large ones), the DOI plot with the LFK index was generated for proportions meta-analysis [12]. The DOI plot is a graphical method to assess the asymmetry for proportions meta-analysis, while the LFK index was created to quantify the asymmetry detected on the DOI plot [12]. The Funnel Plot with the Thomson-Sharp test was used for means meta-analysis [31].

### Classification systems

Several classification systems have been proposed for the IMA branching pattern [1, 41, 43]. The eligible studies in this review did not strictly adhere to one of the proposed classification systems; therefore, we evaluated each study separately and consolidated the results in the following method:

- The IMA bifurcation occurred when two main branches emanated from the main trunk.
- The IMA trifurcation is identified when three main branches originate from the main root.
- The IMA tetrafurcation occurs when four branches are found from the main trunk.

**Fig. 1:**
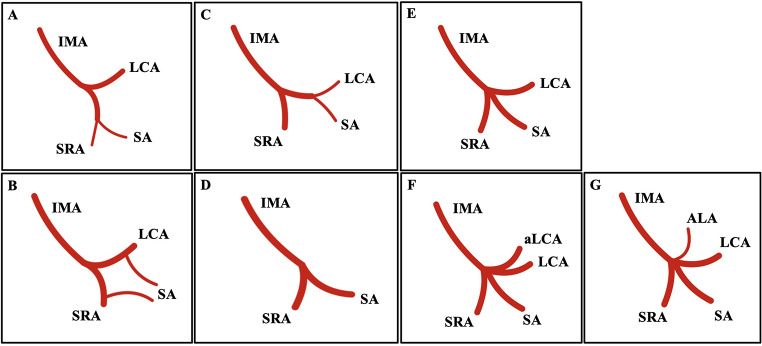
The variant branching patterns of the inferior mesenteric artery (IMA). (**A**, **B**, **C**, **D**) Bifurcation types; (**E**) Trifurcation type; (**F**, **G**) Tetrafurcation types. LCA- left colic artery, SA- sigmoid artery, SRA- superior rectal artery, ALCA- accessory left colic artery, ALA- ascending lumbar artery

These three major groups have a few subtypes based on the specific branching pattern (Fig. 1). Similarly, we have previously classified the CeT variants [35].

## Results

### Studies identification

**Fig. 2:**
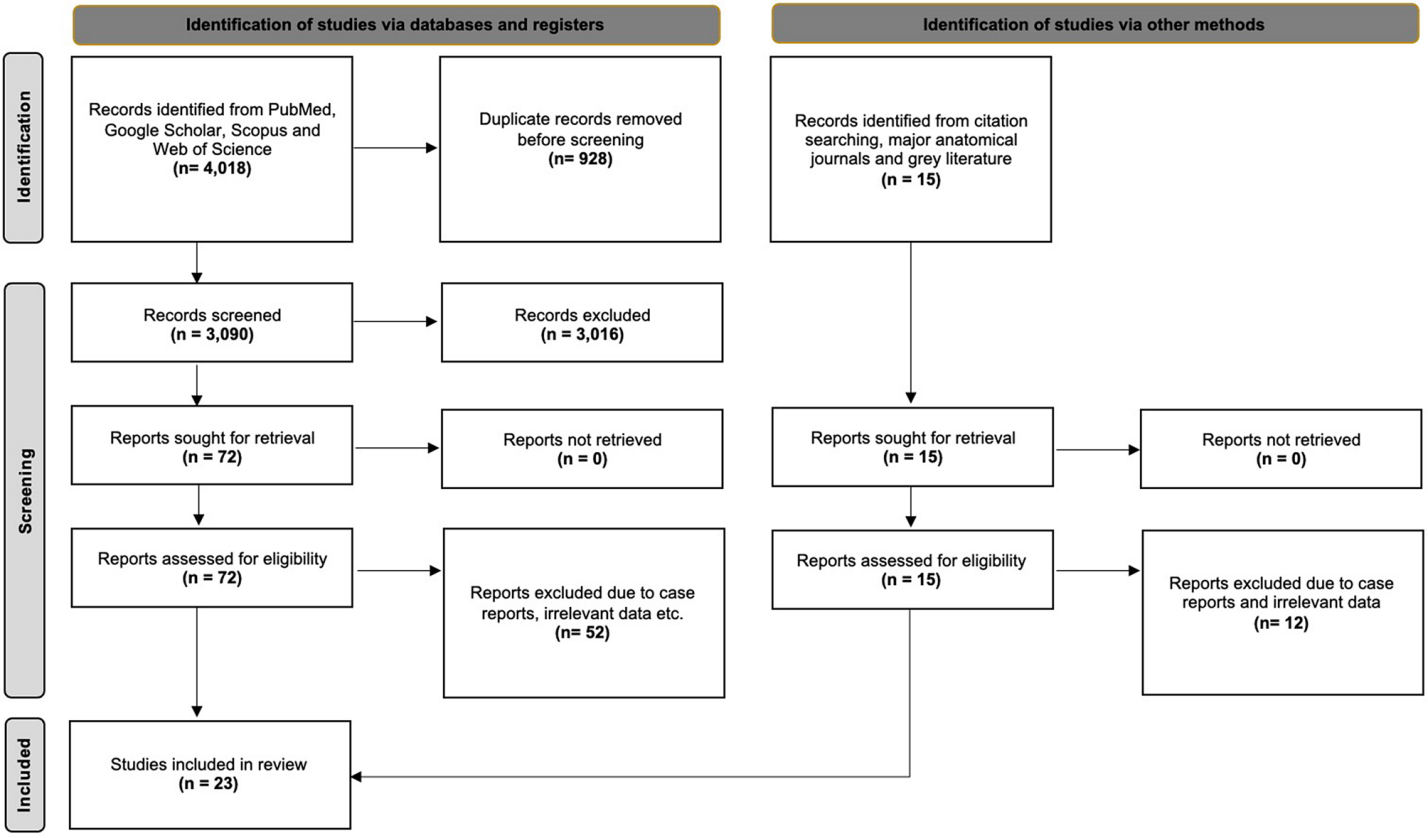
The search analysis flow chart is derived from the PRISMA 2020 guidelines [30]

The database search yielded 4,018 articles exported to Mendeley version 2.10.9 (Elsevier, London). Following the exclusion of duplicate and irrelevant papers through a thorough screening of titles and abstracts, 74 studies proceeded to full-text retrieval and evaluation. Ultimately, 20 studies met the criteria for inclusion in the present systematic review. Additionally, 3 studies were identified through our secondary investigation, encompassing reference searches, grey literature, and hands-on searches for anatomical journals. Consequently, a total of 23 studies were incorporated into our systematic review, which includes a meta-analysis. Figure 2 summarizes the flow diagram illustrating our search analysis following the PRISMA 2020 guidelines.

### Characteristics of the included studies

Twenty-three studies, with a total sample of 3,419 patients, were included. Eleven (11) studies were imaging, ten (10) were cadaveric, and two (2) were surgical. Sixteen studies were based on Asian populations, three were on European populations, two were on American populations, and one was on African populations. The characteristics of eligible studies are summarized in Table 1.

**Table 1:** Characteristics of eligible studies, classified by year of their publication, including their risk of bias assessment according to the AQUA tool [16]

Author (s)	Year	Population	Study type	Age Group	Patients	Risk of Bias
Kahn [17]	1962	America	Imaging	Adults & Children	142	High
Nelson et al. [27]	1988	America	Cadaveric	Adults	50	High
Yada et al. [41]	1997	Asia	Imaging	Adults	344	Low
Kobayashi et al. [20]	2006	Asia	Imaging	Adults	100	High
Ferrari et al. [11]	2007	Europe	Imaging	Adults	60	High
Songur [33]	2010	Asia	Cadaveric	Adults	95	High
Prakash et al. [26]	2011	Asia	Cadaveric	Adults & Children	50	High
Sinkeet et al. [32]	2012	Africa	Cadaveric	NR	55	High
Bertrand et al. [2]	2014	Europe	Imaging	Adults & Children	100	Low
Ubolviroj et al. [37]	2014	Asia	Imaging	Adults & Children	165	High
Koji et al. [21]	2015	Asia	Imaging	Adults	468	Low
Mane et al. [23]	2015	Asia	Cadaveric	Adults	50	High
Gangam et al. [13]	2016	Asia	Cadaveric	Adults & Fetuses	100	High
Nuzhat [28]	2016	Asia	Cadaveric	Fetuses	100	High
Ke et al. [18]	2017	Asia	Imaging	Adults & Children	188	Low
Deepa et al. [8]	2018	Asia	Cadaveric	NR	50	High
Wang et al. [38]	2018	Asia	Surgical	Adults	110	High
Balcerzak et al. [1]	2021	Europe	Cadaveric	Adults	40	High
Ekingen et al. [10]	2021	Asia	Imaging	Adults	238	Low
Zhou et al. [44]	2022	Asia	Imaging	Adults	212	Low
Ding et al. [9]	2024	Asia	Surgical	Adults	200	Low

NR- not reported

### Inferior mesenteric artery topographic details at the level of origin

The origins of the IMA were documented based on the position of the adjacent lumbar vertebrae. The origin at the L2 vertebral level was identified to be 5.27% (*95% CI: 2.42–8.99*). An estimated intervertebral disc level between L2 and L3 vertebrae (L2-L3) indicated a pooled prevalence of 29.79% (*95% CI: 6.41–60.88*). The L3 vertebral level of origin was observed at 70.16% (*95% CI: 56.48–82.25*). The level of IMA origin at the L3-L4 intervertebral disc was recorded at 10.67% (*95% CI: 7.12–14.81*). The origin level at L4 was calculated to exhibit a pooled prevalence of 13.32% (*95% CI: 4.83–24.94*). The level of origin at the L4-L5 intervertebral disc was identified at 19.78% (*95% CI: 14.47–25.67*). Lastly, the level of origin at L5 was estimated to be 3.01% (*95% CI: 0.00-10.89*).

The diameter of the IMA origin was determined to have a pooled mean value of 41.41 mm (*95% CI: 40.37–42.46*). The distance between the IMA and SMA origins was estimated to possess a pooled mean of 64.04 mm (*95% CI: 58.16–69.92*). Additionally, the distance from the origin of the IMA to the AA bifurcation, which leads into the common iliac arteries, was quantified at 37.41 mm (*95% CI: 35.74–39.08*). Finally, the distance between the IMA origin and the LCA origin was noted to have a pooled mean of 40.67 mm (*95% CI: 37.61–42.53*).

### Inferior mesenteric artery standard and rare origins

The IMA is widely described as originating from the AA (typical origin). The IMA origin from the SMA was estimated to have a pooled prevalence of 0.97% (*95% CI: 0.00-3.36%).*

### Variants of the bifurcation branching pattern of the inferior mesenteric artery

The bifurcated branching pattern of the IMA was determined with a pooled prevalence of 63.89% (*95% CI: 49.35–77.26%).* The subgroup analysis, categorized by geographical distribution and study type, did not reveal significant differences (*p* = 0.266 and *p* = 0.075, respectively). The DOI plot illustrated an LFK index of -2, indicating asymmetry. Current literature outlines four distinct bifurcated branch patterns. The predominant pattern involved the IMA bifurcation into the LCA and a common trunk for the SRA and SA, showcasing a pooled prevalence of 46.09% (*95% CI: 39.54–52.71%*). The secondary pattern consisted of the IMA bifurcation into the SRA and a common trunk for the LCA and SA, displaying a pooled prevalence of 27.78% (*95% CI: 17.61–39.22%*). The IMA bifurcation into the LCA and SRA, with the SA originating from both arteries, was noted at 5.41% (*95% CI: 1.48–11.27%*). Furthermore, the IMA bifurcation into the SRA and SA, characterized by the absence of the LCA, was observed in 3.83% of cases (*95% CI: 2.72–5.11%*).

### Variants of the trifurcation branching pattern of the inferior mesenteric artery

An IMA trifurcated branching pattern was estimated, yielding a pooled prevalence of 27.35% (*95% CI: 17.38–38.59%).* The subgroup analysis based on nationality and type of study demonstrated no significant results (*p* = 0.1879 and *p* = 0.5234, respectively). The DOI plot indicated an LFK index of -4.62, suggesting significant asymmetry. It is essential to emphasize that the trifurcated type displayed a unique branching pattern, in which the IMA was divided into the LCA, SRA, and SA.

### Variants of the tetrafurcation branching pattern of the inferior mesenteric artery

The occurrence of a tetrafurcated branching pattern of the IMA is minimally documented in current literature, with a pooled prevalence reported at 11.62% (*95% CI: 7.49–16.46%*). Two types of tetrafurcation have been characterized previously. One specific instance of tetrafurcated IMA, which led to the SRA, SA, LCA, and an accessory left colic artery, was identified with a pooled prevalence of 10.48% (*95% CI: 2.78–21.92%*). Furthermore, occurrences of the typical trifurcation of the IMA (comprising LCA, SRA, and SA) alongside the ascending lumbar artery have been documented only once in existing literature.

## Discussion

This evidence-based systematic review and a meta-analysis evaluate the IMA anatomy. It was determined that the IMA typically bifurcates into the LCA and a common trunk, which subsequently gives rise to the SRA and the SA. Furthermore, the origin of the IMA is located at the level of the third lumbar vertebra. However, the pooled means of several morphometric measurements have been calculated.

### Anatomical considerations of inferior mesenteric artery variants

The level of origin assessed the topographical anatomy of the IMA. Most commonly, the IMA emanated from the L3 level at 70.16% (typical topography), and the most common variant was the level of origin from the L2-L3 intervertebral level at 29.79%. The IMA can be found at a pooled mean distance of 64.04 mm distal to the SMA and 37.41 mm proximal to the AA bifurcation. Interestingly, Balcerzak et al. [1] evaluated if there is a potential correlation between the level of origin and the IMA branching pattern; however, they did not depict a statistically significant result.

In the prevailing body of literature, various classification systems on the branching patterns of the IMA have been systematically reviewed and summarized in the comprehensive analysis by McSweeney et al. [24] and Zeng et al. [43]. Subsequent to these reviews, additional classification systems have been proposed, as Balcerzak et al. [1] indicated. To facilitate a more thorough examination, we developed a classification system based on the division of the IMA into two (bifurcation), three (trifurcation), and four (tetrafurcation) main branches close to the one proposed by McSweeney et al. [24]. Following this categorization, we scrutinized the branching patterns delineated in the existing literature. While this approach does not represent a formal classification system, it provides a relatively straightforward method for categorizing the IMA variants in a unified way. According to research conducted by Zeng et al. [43] and Yada et al. [41], classification remains the most frequently documented aspect of contemporary literature. Yada type I refers to bifurcation involving the LCA and a common trunk for the SRA and the SA. Yada type II indicates a bifurcation into the SRA and a common trunk for both the LCA and the SA. Yada [41] type III includes a trifurcation leading to the LCA, SRA, and SA, while Yada [41] type IV signifies the absence of the LCA with a bifurcation into the SRA and SA. Balcerzak et al. [1] recently outlined five distinct types of IMA. Their classification system introduced two novel types. The first involves the ascending lumbar artery (ALA) originating from the IMA, a phenomenon not previously described. The second distinction is that they did not document instances of LCA absence; instead, they noted that the ascending and descending branches of the LCA originate independently from the IMA [1].

Apart from the IMA main trunk variants, it is also important to highlight each IMA branch morphological variability. Based on the current classification, the LCA can emanate in different patterns: from the IMA main trunk, in a common trunk with SA, or be absent. Cirocchi et al. [5] described a few variants of the LCA in their systematic review with meta-analysis. They identified the LCA absence in 4.1%, the “spread-out” origin of the LCA in 49%, and the “fan-shaped” origin of the LCA in 51% of patients. Furthermore, we identified a few variants of the SA, such as the origin in a common trunk with the SRA, a common trunk with the LCA, or from the IMA main trunk. Cirocchi et al. [6] recorded several variations of the SA in their meta-analysis. The SA originating from the SRA was identified at 49.67%, the origin from the LCA was determined at 25.26%, and multiple SAs emanating from SRA and LCA were observed at 0.18%. In comparison, the origin directly from the IMA was recorded at 13.26%. Nevertheless, they described that the SA could vary from one to five branches, with the most prevalent being the three branches. Cirocchi et al. [6] highlighted that the SA depicts a significant morphological variability. The SRA could be characterized as the most contact IMA branch because it does not represent substantial variability.

The morphological variability of the branching pattern remains ambiguous; however, genuine anatomical variations have been insufficiently documented. We noted a pooled prevalence of 0.97% concerning the origin of the IMA from the SMA, which has also been mentioned in several case reports in the current literature [29, 42]. Furthermore, the testicular artery was identified as originating from the IMA during angiographic procedures [25]. Kim and Han [19] reported another unique instance during dissection, observing the absence of the IMA while the typical branches from the middle colic, inferior pancreaticoduodenal, and SMA were present.

### Surgical considerations of inferior mesenteric artery variants

**Fig. 3:**
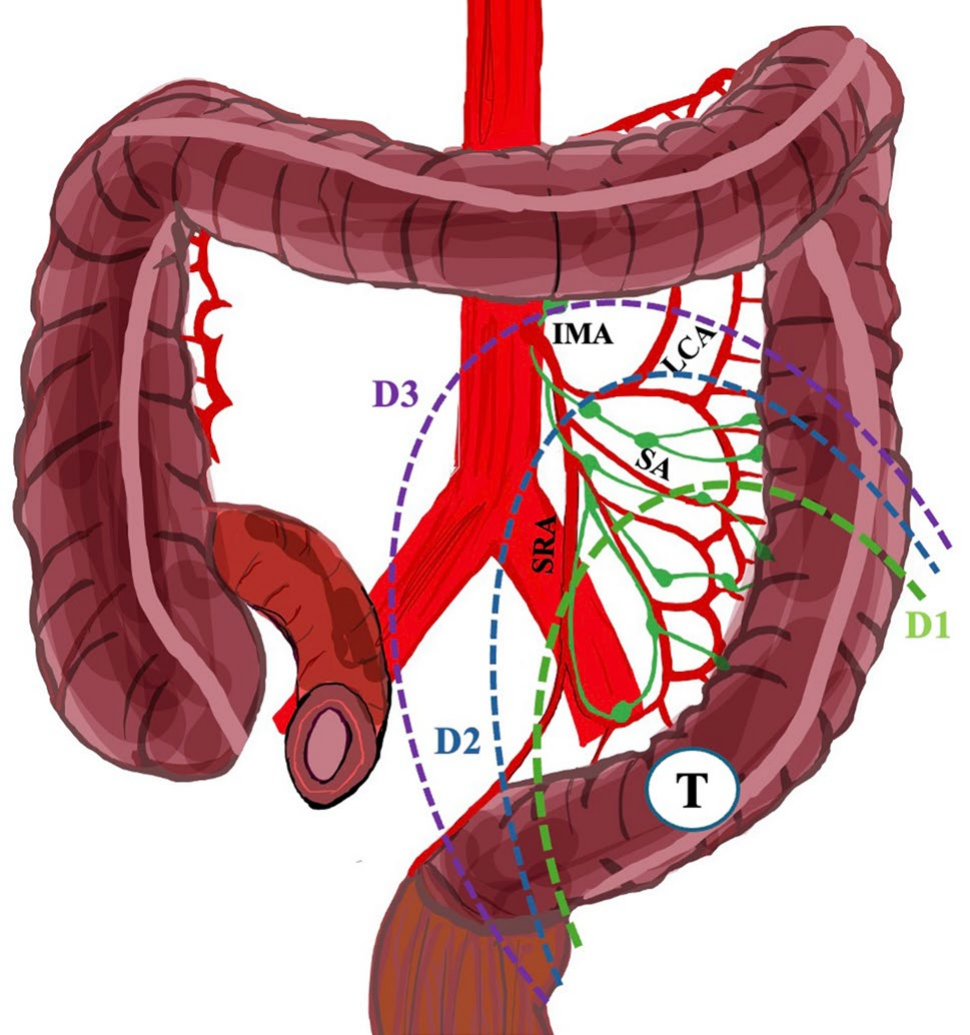
The dissection of lymph nodes is based on ligation of the inferior mesenteric artery (IMA) when a tumor (T) is present at the rectosigmoid junction. LCA stands for left colic artery; SRA is the superior rectal artery; and SA is the sigmoid artery

The IMA is recognized as the most vital anatomical landmark in colorectal surgery; therefore, a thorough understanding of its surgical anatomy is essential [43]. Specifically, the IMA is crucial in total mesorectal excision, lymph node dissection near its root, and preserving pelvic autonomic nerves. In managing rectal cancer, lymph node dissection must involve the main trunk of the IMA. A significant debate persists regarding the optimal level for IMA ligation during left colon or rectal resection—whether high or low ligation is preferable—and whether the LCA should be preserved [43]. High ligation refers to ligating the main IMA trunk without preserving the LCA. In contrast, low ligation refers to ligation in the LCA region involving lymph node dissection at the IMA root and preservation of the LCA (Fig. 3). In both cases, the IMA’s origin and branching pattern are critically important, as variations may affect the chosen procedure (high or low ligation) [43]. The intraoperative identification of the precise IMA origin from the anterior surface of the aorta will aid in the dissection during lower anterior resection for rectal cancer using a medial-to-lateral approach to ensure maximum harvesting of regional lymph nodes. The current meta-analysis illustrated the pooled mean distance from the IMA origin to other key and constant vessels (SMA, AA bifurcation). From a surgical standpoint, the lower border of the duodenum, along with visualization of the IMV root, serves as an anatomical landmark to pinpoint the exact IMA origin from the aorta. Proper high ligation entails ligating the main trunk of the IMA, positioned 1 to 2 cm away from the abdominal aorta’s origin, to ensure a more radical oncological lymphadenectomy (D3) while preventing injury to pelvic autonomic nerves (Fig. 3).

The current meta-analysis indicates that, in most cases, the IMA bifurcates with a pooled prevalence of 63.89%. Low ligation with LCA preservation is technically manageable, yielding favorable outcomes [22]. When tumor and patients’ characteristics allow it, preserving the IMA and the LCA may be crucial to facilitate a better vascularization of the descending colon for a safer anastomosis. However, surgeons should remember that a disadvantage of the low ligation with LCA preservation is the insufficient repositioning of the descending colon for the anastomosis with the rectal stump due to short mesocolon, resulting in inappropriate tension for anastomosis. In such scenarios, the ligation of IMA and LCA might be inevitable. When high ligation should be performed, vascularization from the marginal artery of Drummond should be adequate to supply the anastomosis [5]. Surgeons should also acknowledge that the SA can emanate from a common trunk with the LCA (*27.78% pooled prevalence*) or directly from the LCA (*5.41% pooled prevalence*); These anatomical possibilities could also play a role in preserving the LCA. Our findings demonstrate that the pooled mean distance between the IMA and LCA is approximately 40.67 mm, a parameter that may have significant implications for determining the appropriateness of high or low ligation.

**Fig. 4:**
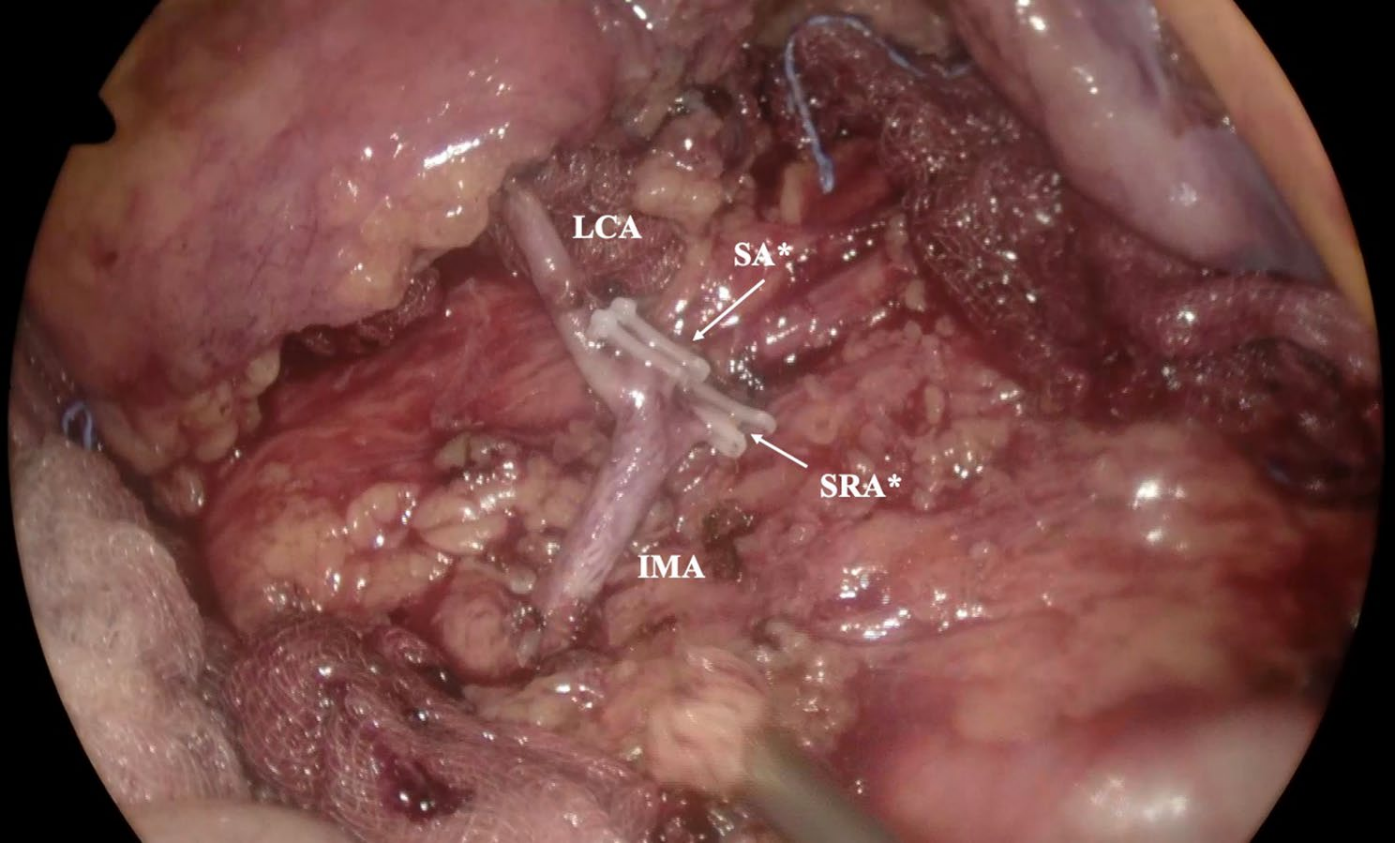
Intraoperative impression of an inferior mesenteric artery (IMA) trifurcation during laparoscopic lower anterior resection for mid-rectal cancer, involving low ligation of IMA while preserving the left colic artery (LCA). The branches of the sigmoid artery (SA) and superior rectal artery (SRA) are selectively ligated (*). Personal Archive of Ass. Prof. Orestis Lyros

Low ligation with LCA preservation is recommended in patients exhibiting IMA trifurcation, which represents a pooled prevalence of 27.35%. High ligation could impede LCA blood flow, resulting in inadequate perfusion of the descending colon and distal anastomosis. Nevertheless, as the IMA is trifurcated into three branches, high ligation will result in the loss of all branches. In these cases, the descending colon/colonic conduit and colorectal anastomosis perfusion depends solely on the marginal artery of Drummond, while the splenic flexure and part of the descending colon would receive blood supply from the arches of the middle colic artery. However, all these details should be carefully addressed by the surgeon to achieve the best result [43] (Fig. 4).

Although IMA tetrafurcation infrequently occurs, with a pooled prevalence of 11.62%, surgeons must recognize that additional arteries may arise from the IMA, including the ALA; thus, high ligation may result in inadequate blood supply [43]. Another branching pattern of the IMA tetrafurcation, which includes the accessory LCA, could be advantageous as it provides extra blood supply to the LCA territory.

Furthermore, tumor staging is critical to colorectal cancer treatment, affecting treatment selection and the ligation technique (high or low, with or without LCA preservation) [43]. The results of this meta-analysis demonstrate that the IMA shows significant morphological variability, which requires preoperative documentation through computed tomography angiography or intraoperative bowel perfusion evaluation using Indocyanine Green Fluorescence-guided surgical techniques [14]. Additionally, the IMA’s origin level, usually located at the level of L3 according to adjacent lumbar vertebrae, may also represent an essential detail for interventional procedures.

### Limitations

The present evidence-based meta-analysis delineates several limitations. A substantial degree of heterogeneity was observed among the statistical results, and the DOI plots indicated an asymmetry that implies a potential small-study effect. Numerous studies have been identified as exhibiting a high risk of bias, as evaluated by the AQUA tool. Nonetheless, these findings are congruent with observations made in anatomical meta-analyses [16]. The creation of funnel plots for pooled means proved unfeasible, given that morphometric parameters were investigated in fewer than five studies [31]. Regrettably, the number of studies accessible for each parameter included in the current analysis was inadequate to conduct subgroup analyses based on nationality and study type.

## Conclusions

The present systematic review, accompanied by a meta-analysis, evaluates the IMA’s branching pattern, origin level, and morphometric characteristics. It is observed that the IMA predominantly bifurcates into the LCA and a common trunk, which further supplies the SRA and the SA, with its origin placement noted at the third lumbar vertebral level. Anatomical variations, particularly regarding the branching configuration, are frequently documented and are of substantial surgical relevance. Several classification systems were proposed in the current classification, while the current meta-analysis aimed to unify these results. Preoperative familiarity with the IMA anatomy is critical as it influences the selection of surgical procedures, specifically in determining whether to perform high or low ligation, with or without the preservation of the LCA, in the context of colorectal cancer treatment.

## Electronic supplementary material

Below is the link to the electronic supplementary material.

Supplementary Material 1

## Data Availability

No datasets were generated or analysed during the current study.
