# Supplementary material for: The anatomy of the inferior mesenteric artery: a systematic review with meta-analysis
Source: Surg Radiol Anat. 2025 May 24;47(1):144. doi: 10.1007/s00276-025-03657-1 (PMC12103349; doi:10.1007/s00276-025-03657-1)
Supplement: Supplementary file 1 — Supplementary Material 1 [file 276_2025_3657_MOESM1_ESM.docx]

**Supplementary Materials**

***Supplementary Figure 1. Forest plot for the bifurcation pattern.***
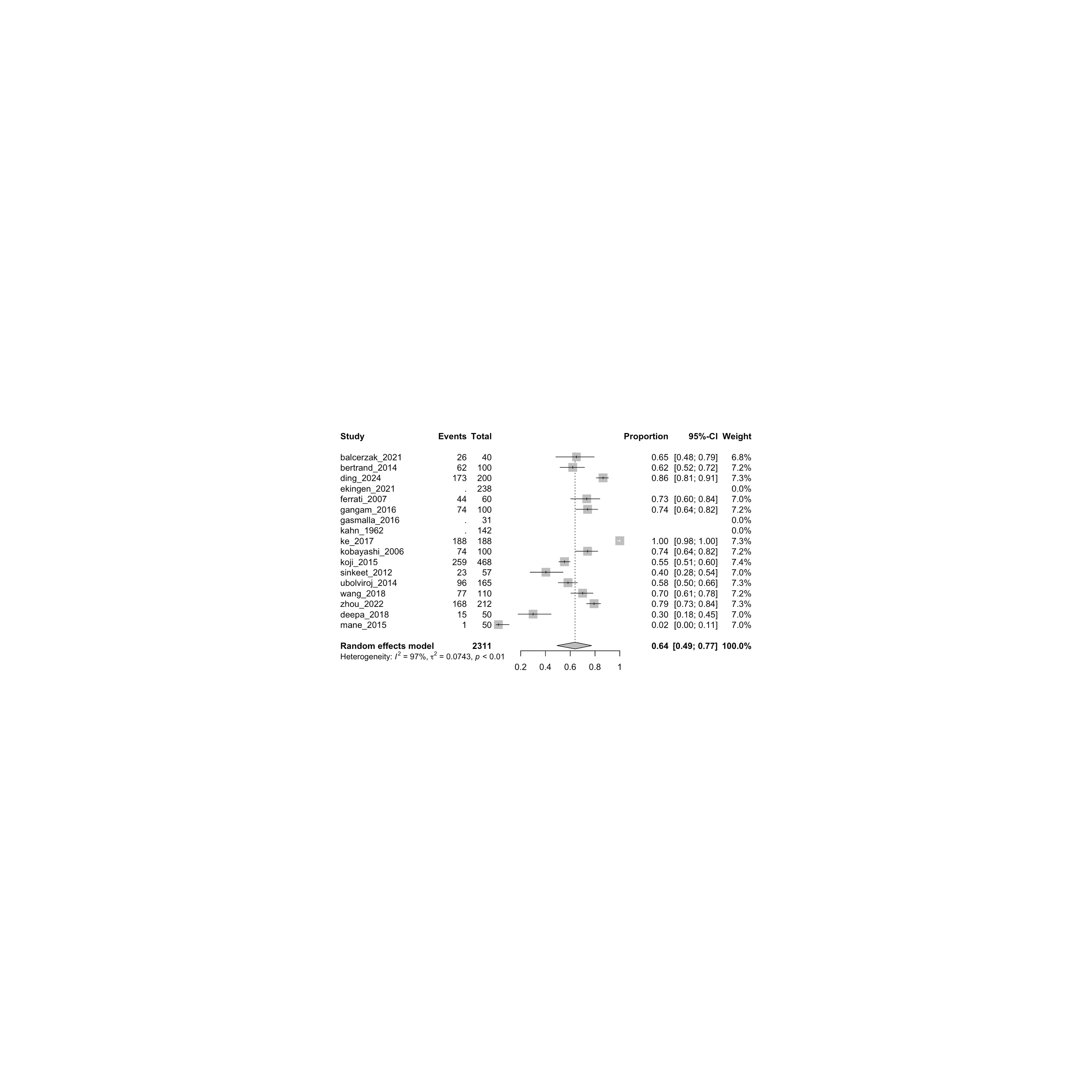


***Supplementary Figure 2. Forest plot for the trifurcation pattern.***


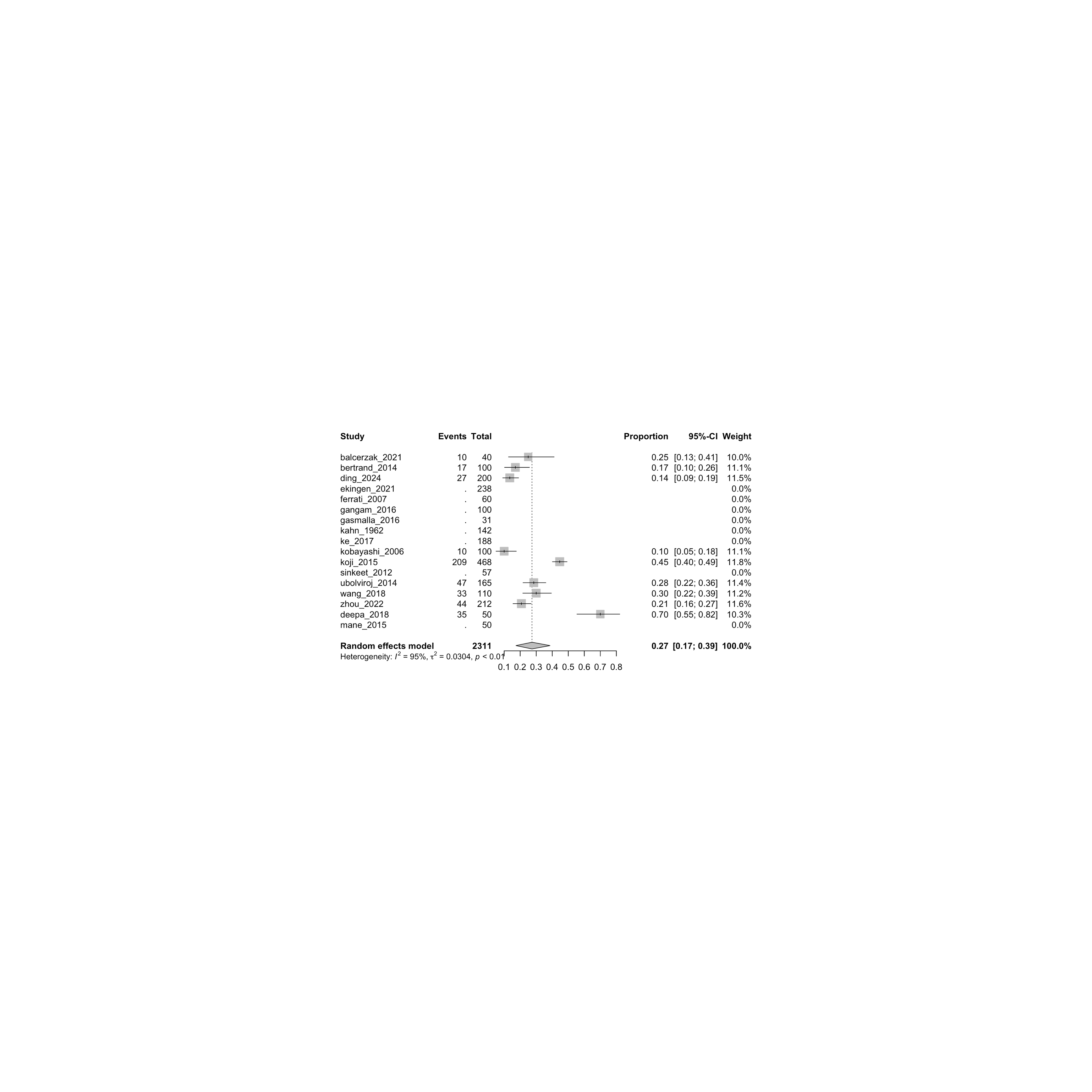


***Supplementary Figure 3. Forest plot for the tetrafurcation pattern.***


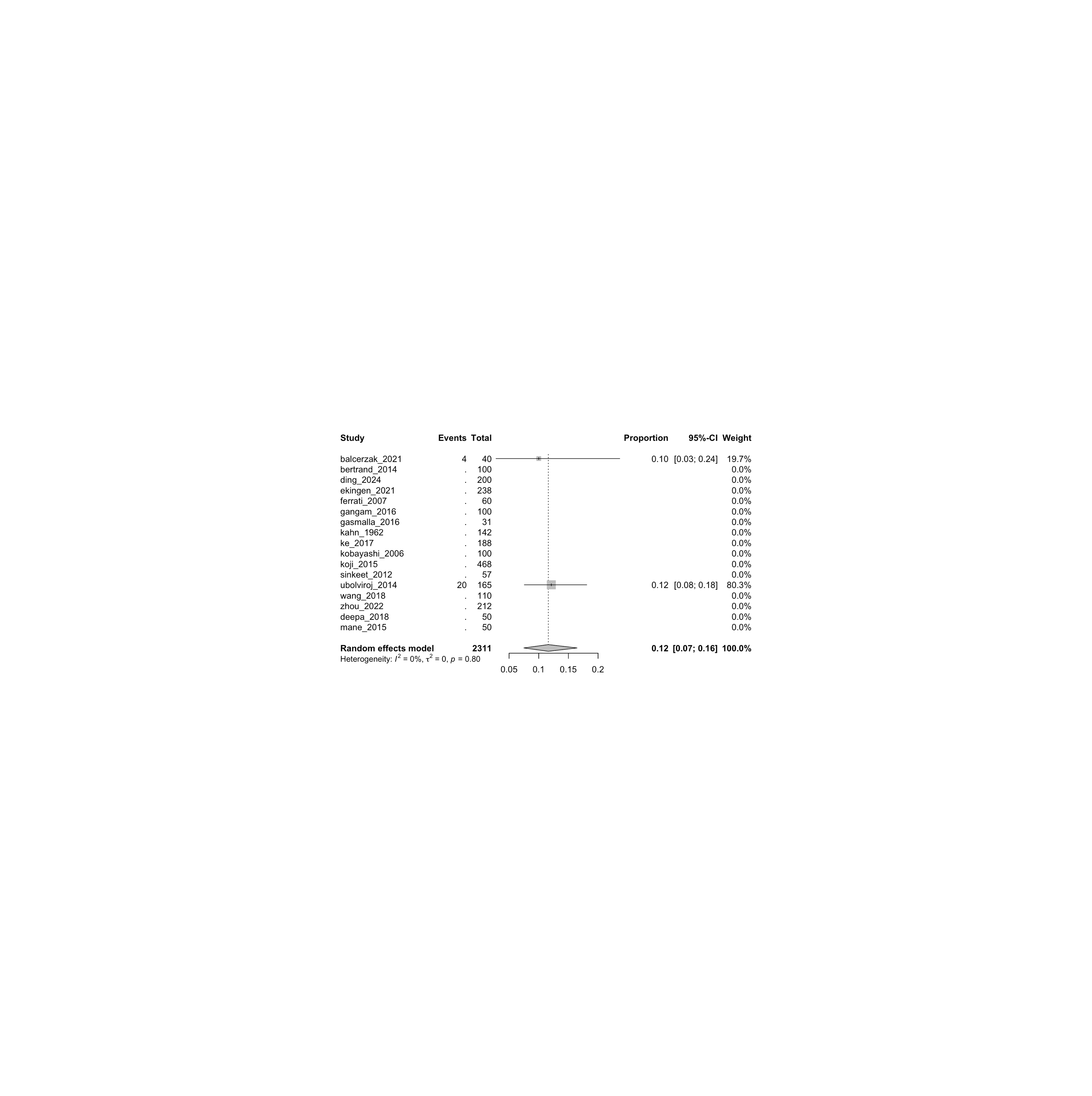


***Supplementary Figure 4. DOI plot for the bifurcation pattern pooled prevalence.***

***
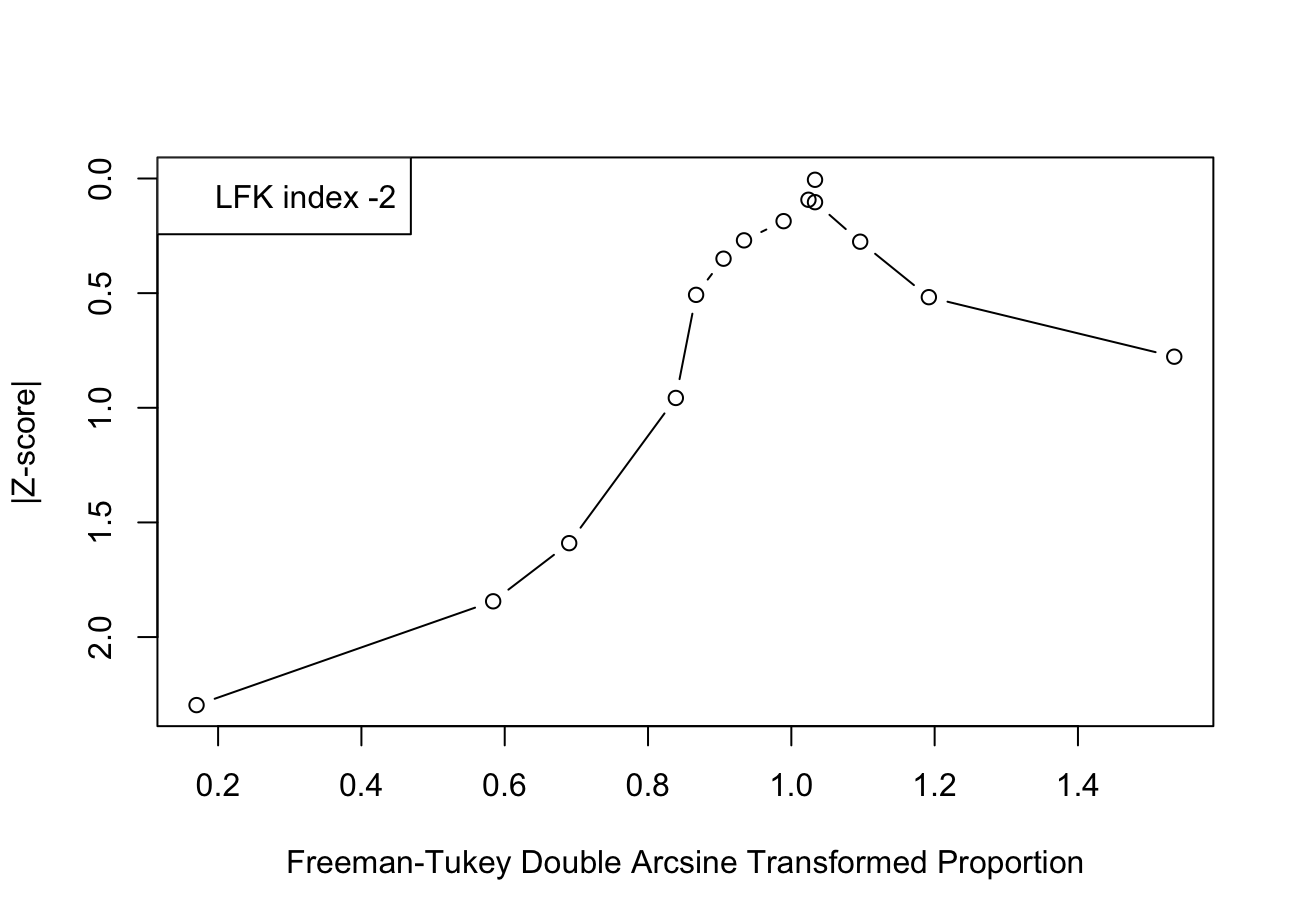
***

***Supplementary Figure 4. DOI plot for the trifurcation pattern pooled prevalence.***

***
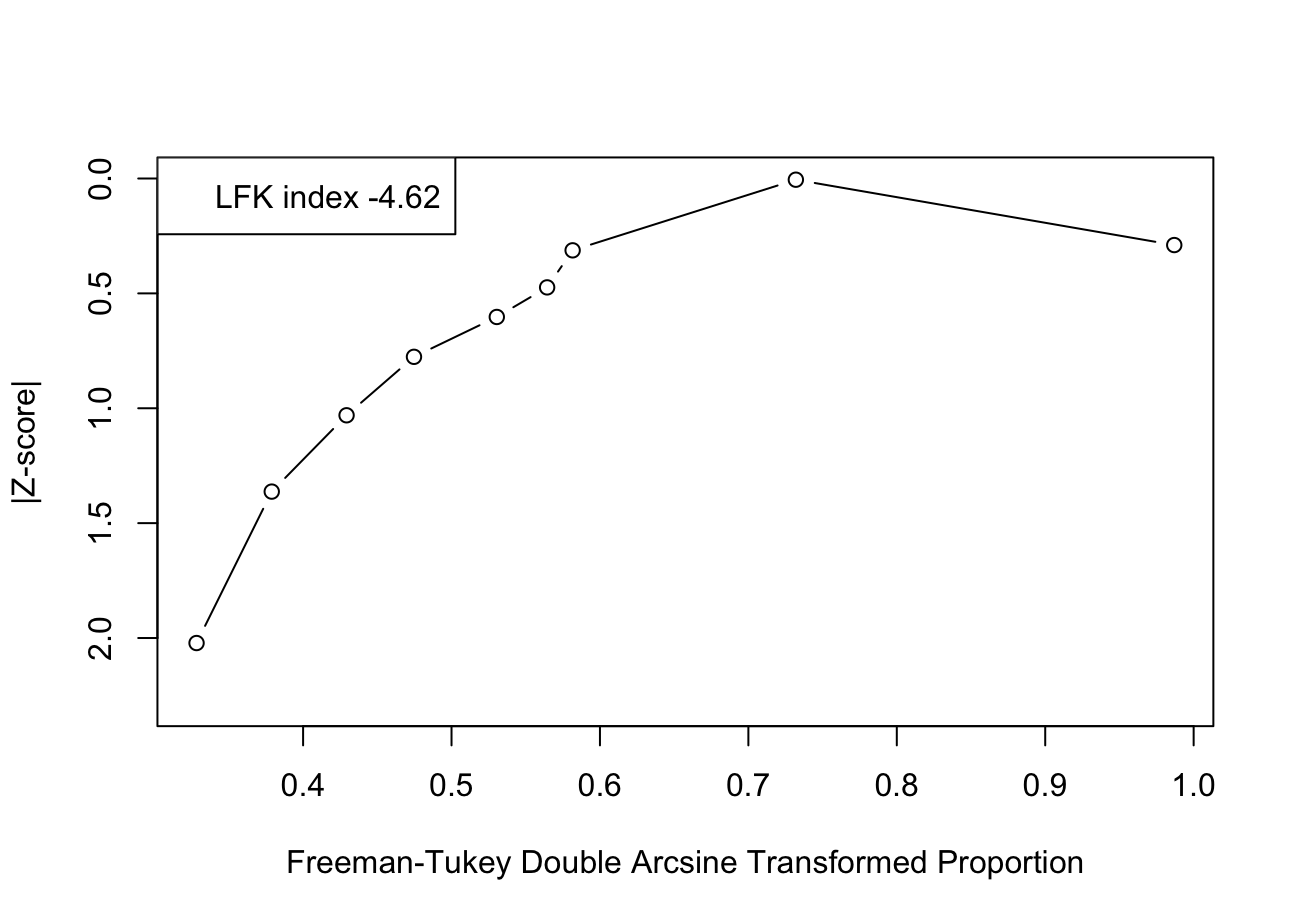
***
